# Multidisciplinary Management of Infantile Hypophosphatasia Resulting in Radiographic and Clinical Improvement: A Case Report

**DOI:** 10.7759/cureus.25426

**Published:** 2022-05-28

**Authors:** Leeann Qubain, Pamela Smith, Neeraj Vij, Mohan Belthur

**Affiliations:** 1 Department of Orthopaedic Surgery, University of Arizona College of Medicine, Phoenix, USA; 2 Department of Endocrinology, Phoenix Children's Hospital, Phoenix, USA; 3 Pediatric Orthopaedics, Phoenix Children's Hospital, Phoenix, USA; 4 Pediatric Orthopaedics, University of Arizona College of Medicine, Phoenix, USA

**Keywords:** pediatric trauma, evidence-based medicine, endocrinopathy, enzyme replacement therapy, pediatric orthopedics, multidisciplinary care

## Abstract

Hypophosphatasia (HPP) is a rare genetic condition that can manifest from the prenatal period to adulthood. Clinical presentation is characterized by six major forms. HPP can be complex and debilitating. A two-year-old male with a past medical history of HPP presented to our emergency room with a non-displaced supracondylar fracture after minor trauma. Non-accidental trauma was considered in addition to inadequate medical control of his HPP. He was referred to our multidisciplinary clinic and asfotase alfa was increased to an appropriate dose. A multidisciplinary approach is the standard of care for the management of children with HPP, allowing for routine evaluation by tertiary specialists. This includes medication dosing surveillance with serum studies and imaging. Enzyme replacement therapy, appropriately dosed by considering weight and laboratory values, may reduce orthopedic complications. A multidisciplinary team's surveillance of patients with HPP ensures proper medication management, decreases the likelihood of bony injury and encourages continued patient follow-up.

## Introduction

Hypophosphatasia (HPP) is a rare, heritable bone condition caused by variants in the alkaline phosphatase (ALPL) gene on chromosome one (1p36.12) resulting in under-mineralization of the skeleton and teeth. It is estimated that one in 100,000 live births is affected by HPP in North America [1]. Homozygous or compound heterozygous mutations in ALPL result in moderate to severe phenotypes of HPP [2]. ALPL codes for tissue non-specific alkaline phosphatase (TNSALP). TNSALP de-phosphorylates inorganic pyrophosphate (PPi), an inhibitor of bone mineralization by osteoblasts and chondrocytes. Clinical presentation is a spectrum of severity. Individuals with HPP may be asymptomatic carriers of the condition or present with early loss of deciduous teeth only (before age 5), exhibit rickets or osteomalacia with susceptibility to fracture with poor growth and development in childhood, or exhibit muscle weakness, chronic pain, respiratory failure, or even death in utero or early infancy [3].

Six clinical forms of HPP have been described: odonto-HPP, benign perinatal, infantile, childhood, adulthood, and perinatal lethal HPP [4]. The benign form, odonto-HPP, is the most common and causes early loss of deciduous teeth without skeletal abnormalities or other symptoms. The perinatal lethal form is extremely severe and typically causes fetal demise or death after birth. Milder forms in adolescence and adulthood can be missed due to non-specific complaints like arthralgias, myalgias, metatarsal stress fractures, and chronic bone pain [5]. Significant complications include seizures in infancy, failure to thrive, long bone deformities, limb length discrepancy, short stature, ectopic calcifications of the eyes, nephrocalcinosis, and cardiovascular disease in adulthood [4,5].

Diagnosis of HPP includes a history of recurrent fragility fractures, short height, low body weight, low serum alkaline phosphatase (ALP), and “tongues of radiolucencies” on radiography [4]. The severity of HPP directly correlates with the reduction of measured serum ALP, adjusted for age and sex. As a result, ALP enzyme substrates build up in the system, including pyridoxal 5’-phosphate (PLP), phosphoethanolamine (PEA), and PPi. Increased PPi inhibits bone mineralization by preventing calcium and phosphorus deposition. Other blood and urine studies that support the diagnosis are elevated urinary calcium (with or without elevated serum calcium), high-normal to elevated serum phosphorus level, and elevated serum Vitamin B6 or PLP. Micro-Computed Tomography analysis of the bone may show hypo-mineralization, abnormal trabecular architecture in the femoral epiphyses, and trabecular and cortical thinning. Genetic testing is not required for diagnosis but offers confirmation and allows for appropriate genetic counseling for families [5].

Recently, enzyme replacement therapy (ERT) with asfotase alfa has been approved for HPP in the pediatric population [6]. ERT has been shown to reduce mortality during the first year of life from ∼97% in perinatal cases and ~60% in infantile cases to approximately 10% overall [2]. Conventional wisdom dictates that asfotase alfa be dosed based on patient weight. Calcium and vitamin D supplementation may be also needed to promote maximal bone mineralization [7].

In this report, we present a case of a young child with inadequate medical management of HPP based on conventional wisdom leading to a non-displaced supracondylar fragility fracture. After initiation of multidisciplinary management and careful dosing adjustment, normalizing laboratory values, bony healing, and the absence of recurrent fragility fractures were noted.

## Case presentation

Initial presentation

A two-year-old male presented with acute left elbow pain after striking the flexed elbow against a nightstand. The patient’s mother denied open injury, neurologic symptoms, or concurrent injury. Medical history was pertinent for infantile hypophosphatasia diagnosed at birth and he was currently receiving asfotase alfa 14 milligrams subcutaneously three times per week (4.5 mg/kg/week).

The patient was 9.4 kg (<1st percentile) and 80.4 cm (4th percentile). Physical exam demonstrated mild joint swelling but no visible deformity. There was palpable tenderness over the elbow. There was no evidence of neurovascular defects or concurrent injury.

Investigations

Non-accidental trauma and inadequate control of the hypophosphatasia were both considered. The patient underwent laboratory investigations including a comprehensive metabolic panel and urine calcium to creatinine ratio. Radiography was also performed.

Laboratory investigations revealed an elevated serum ALP, an elevated aspartate aminotransferase (AST), a normal alanine transaminase (ALT), and an elevated urine calcium to creatinine ratio (Table 1).

**Table 1:** Laboratory values on initial presentation and after dosing regimen change This chart depicts the standard laboratory values used in monitoring hypophosphotasia. A decrease in the initially high aspartate aminotransferase and urine calcium to creatinine ratio can be seen by 30 months after the dosing regimen change.

Laboratory Value	Units	Initial Presentation	Six Months After Dosing Regimen Change	30 Months After Dosing Regimen Change	Reference Range
Alkaline Phosphatase	IU/L	9,500	13,020	12,000	131-387
Aspartate Aminotransferase	IU/L	67	62	51	10-50
Alanine Transaminase	IU/L	18	20	32	5-41
Urine Calcium to Creatinine	mg/g	883	363	268	20-500

**Figure 1:**
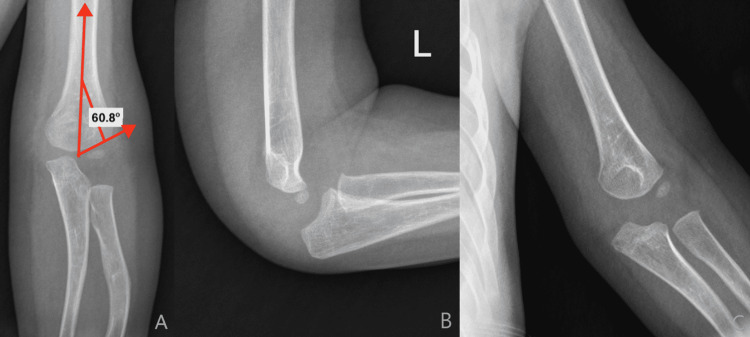
Radiography on initial presentation AP (Panel A), Lateral (Panel B), and Oblique (Panel C) views of the left elbow demonstrating a Baumann’s angle of 60.8°, subtle buckling of the medial supracondylar line, and a moderate effusion consistent with a nondisplaced supracondylar fracture. A plastic deformation of proximal radius can also be noted on the AP.

Radiographs demonstrated a non-displaced supracondylar fracture of the left distal humerus and tongues of radiolucency throughout the distal humeral metaphysis (Figure 1).

Initial management

Non-operative management was offered given his non-displaced fracture. He was placed in a long-arm cast for three weeks that was then removed and repeat imaging was performed. His elbow fracture healed uneventfully.

Complications

**Figure 2:**
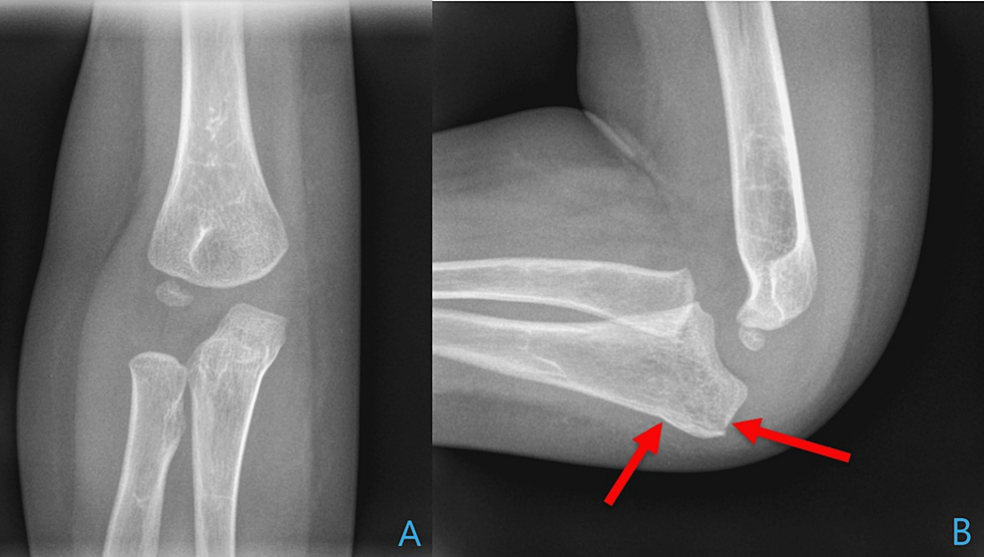
Radiography of the subsequent injury AP (Panel A) and Lateral (Panel B) of the right elbow demonstrating a radiolucent line parallel to the dorsal aspect of the proximal ulna with a cortical break consistent with an incomplete olecranon fracture.

Four months after the initial injury, he presented with a second minor trauma. His mother was unclear as to the mechanism of injury but denied major falls or trauma. Non-accidental trauma was considered, but clinical suspicion was low given his medical history. His mother denied open injury, neurologic symptoms, or concurrent injury. Radiographs revealed a non-displaced right olecranon fracture (Figure 2).

Multidisciplinary management

**Figure 3:**
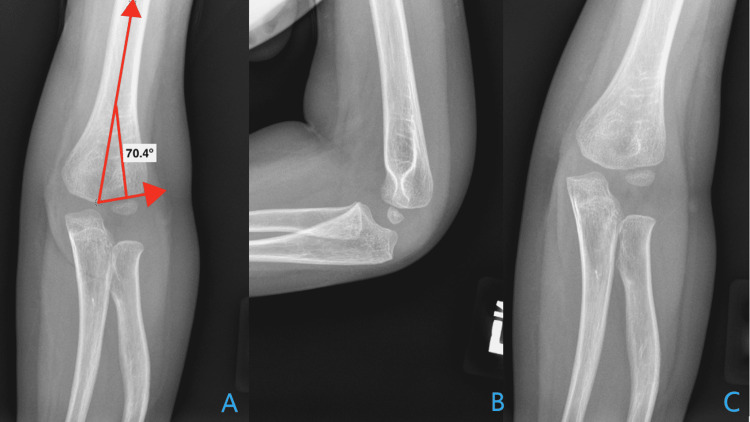
Radiography at the 18 month follow-up AP (Panel A), Lateral (Panel B), and External Oblique (Panel C) views of the priorly fractured left elbow 18 months after injury demonstrating a restoration of Baumann’s angle and an absence of the posterior fat pad sign indicative of normal healing.

Given his recurrent fragility fractures, he was referred to a skeletal health clinic which includes orthopedics, genetics, and endocrinology. In the clinic, his asfotase alfa dose was increased to 40 (mg) subcutaneously three times a week (12.8 mg/kg/week) and he was routinely followed. Serial radiographs were obtained to monitor the healing of his initial elbow fractures (Figure 3).

Serial biochemical studies were obtained to monitor his bone health.

Follow-up

Six months after increasing asfotase alfa, biochemical studies demonstrated favorable changes: a decrease in AST, a normal ALT, and a normal urine calcium to creatinine ratio. However, the patient maintained elevated ALP (Table 1).

After thirty months, studies demonstrated further favorable changes: a continued decrease in AST, a maintained normal ALT, a maintained normal calcium to creatinine, and a decreasing, though still elevated, ALP (Table 1). The family reported no interval bone pain and resolution of prior injuries.

## Discussion

We present a case of inadequate control of infantile hypophosphatasia resulting in recurring fragility fractures. Close examination of the fractures revealed a subtle concurrent buckle fracture, an obvious plastic deformation of the ulna, and radiolucent tongues consistent with endocrinopathy. Following referral to our multidisciplinary clinic, proper dosing of asfotase alpha led to an improvement in laboratory values, fracture healing, and the absence of further fragility fractures at thirty months. 

Asfotase alfa was approved in 2015 for pediatric-onset HPP and has been shown to improve muscle strength, mobility, and long-term outcomes when used at correct doses [8,9]. ERT has demonstrated good utility in improving survival from one to five years when used for perinatal and infantile HPP [10]. Dosing of asfotase alfa for pediatric and adult patients depends on the HPP subtype and clinical presentation. In children, the standard dose is up to six mg/kg/week [10]. In our patient, his dose was increased from 14 mg to 40 mg three times a week (4.5 mg/kg/week to 12.8 mg/kg/week).

Asfotase alfa has side effects that should be heavily considered before starting medication. Adverse effects include lipodystrophy, ectopic calcifications, hypersensitivity, skin discoloration, and local injection site rejections [10]. A more serious, less common side effect is hepatitis. Therefore, those with mild HPP symptoms should be placed on a custom dose regimen. Dosing should be reduced to two mg/kg once per week once an adequate response is achieved in pediatric patients [8]. Meanwhile, severe cases such as perinatal or infantile forms may require higher doses - upwards of 9 mg/kg/week. Children and adults with symptomatic HPP who are without ERT can experience progressive muscle weakness and bone density deterioration [11].

Children should be monitored by a comprehensive skeletal health clinic. This allows for comprehensive surveillance of medications, potential complications, and any need for surgical or other therapeutic interventions [12]. Specialties can include ophthalmology, neurosurgery, endocrinology, genetics, orthopedics, nutrition, and dentistry [12]. Monitoring varies depending on disease severity and age but includes serum and urine biochemical studies and radiographs to assess for rachitic changes, “tongues of radiolucency”, metatarsal fractures, and pseudofractures characteristic of HPP [12]. Biochemical studies and imaging are routinely obtained until adult height is achieved. In cases of concomitant Chiari 1 malformation or syringomyelia, monitoring should be pursued indefinitely [7].

Due to delayed referral to our multidisciplinary clinic and loss of follow-up, our patient’s ERT dosing regimen was not appropriately monitored and adjusted, resulting in recurrent fragility fractures. Following dose correction, fracture frequency decreased and he demonstrated normal bone healing (Figure 3).

## Conclusions

Our case highlights several important teaching points. Early diagnosis of clinically significant HPP allows for proper intervention with ERT, vitamin and mineral supplementation, physical and occupational therapies, as well as for surgical assessments by orthopedics and neurosurgery. Secondly, proper management may include dosing adjustment based on laboratory values in the setting of a comprehensive skeletal health clinic; however, the pros of improved bone health need to be weighed against the serious side effects of the medication. Appropriate management can prevent bone demineralization and fragility fractures in symptomatic HPP. Lastly, a multidisciplinary approach should be implemented as early as possible to promote comprehensive surveillance, ensure appropriate medication management, and ensure continued follow-up.
